# First record of *Aphelenchoides stammeri* (Nematoda: Aphelenchoididae) from Turkey

**DOI:** 10.21307/jofnem-2019-070

**Published:** 2019-10-14

**Authors:** Mehmet Dayi, Ece B. Kasapoğlu Uludamar, Süleyman Akbulut, İ. Halil Elekcioğlu

**Affiliations:** 1 Forestry Vocational School, Düzce University, Düzce, Turkey; 2 Department of Plant Protection, Faculty of Agriculture, Çukurova University, Adana, Turkey; 3 Department of Forest Engineering, Faculty of Forestry, İzmir Katip Çelebi University, İzmir, Turkey

**Keywords:** *Pinus*, Survey, Vector, *Aphelenchoides stammeri*, Detection, Morphology, Diagnosis

## Abstract

In previous surveys for the investigation of *Bursaphelenchus xylophilus* in Turkey, several *Bursaphelenchus* species, except *B. xylophilus*, were detected. To determine the insect vectors of these previously reported *Bursaphelenchus* species, trap trees of each pine species (*Pinus brutia* and *P. nigra*) were cut and kept in the field from April to September in 2011. *Aphelenchoides stammeri* was isolated from wood chip samples of *P. nigra* from Isparta (Sütçüler). *A. stammeri* was first identified by using morphological features and allometric criteria of females and males. In addition, molecular analysis using sequences of 18S and 28S ribosomal (rRNA) genes of *A. stammeri* was performed. This is the first report of *A. stammeri* from Turkey.

Pine wilt disease, caused by the pinewood nematode, *Bursaphelenchus xylophilus* (Steiner and Buhrer, 1934; Nickle, 1970) (Nematoda: Parasitaphelenchidae), has been leading to extensive damage in pine forests of eastern Asia (Webster, 1999). After detection of the nematode in Portugal (Mota et al., 1999), the scientific interest in this wood-inhabiting group of nematodes and other components of the pine wilt disease (host tree and vector insects) has increased in European countries. Although the European Union imposed specific measures to control, eradicate, and prevent the spread of the pinewood nematode into uninfected regions within the European Union borders, the nematode was reported from Spain (Abelleira et al., 2011; Robertson et al., 2011). Recently, *B. xylophilus* was also reported from a new host tree (*Pinus nigra*) in Portugal (Inacio et al., 2015).

Turkey has a very important geographical location between Europe and Asia, which increases the possibility of introduction of *B. xylophilus* from infested regions. Therefore, the survey of *B. xylophilus* has been periodically carried out in different geographical regions of Turkey since 2003.

In 2011, a new project was started to collect the possible insect vectors of *Bursaphelenchus* Fuchs species in some forest sites of Turkey. During this study, potential insect vectors and wood samples were collected using different techniques. In addition to *Bursaphelenchus* species, a species of *Aphelenchoides* Fischer was found in wood samples of *Pinus nigra* Arnold tree taken from the forest site of Isparta Regional Forestry Directorate, the southwest part of Turkey.

In this study, *Aphelenchoides stammeri* was identified as a first record for Turkey by using morphological characteristics and molecular techniques and compared with the original descriptions.

## Materials and methods

### Nematode collection and isolation

In 2011, a total of five trap trees (mean 20 m in length and 30 cm in diam.) of each pine species (*Pinus brutia* Ten. in İzmir and *P. nigra* in Isparta) were prepared to catch the possible insect vectors of *Bursaphelenchus* species in forest sites of İzmir and Isparta Regional Forestry Directorates, respectively. Trap trees composed of healthy trees without any insect and nematodes presence, based on pre-sampled results, were placed at specific locations (Bergama in İzmir and Sütçüler in Isparta) before the beginning of the flight period of insects (early February), and they were kept in the field until September for oviposition of insects. The specific locations of trap trees were selected according to the results of *Bursaphelenchus* survey studies carried out in 2007 and 2008 in which the presence of *Bursaphelenchus* species was previously reported from these locations. Approximately 300 g of wood chip samples were taken from different points of the trap trees by using a borer, put into sealed plastic bags and sent to the laboratory to check for the presence of nematodes under a light microscope (Olympus SZX12, Olympus BX-51). All nematodes belonging to the genera of *Bursaphelenchus* and *Aphelenchoides* were cultured on *Botrytis cinerea* Pars., growing on malt agar, and incubated at least for 2 wk at 25°C.

The nematode specimens were killed by heat, fixed in TAF (7 ml formalin (formaldehyde % 40) + 2 ml triethanolamine + 91 ml pure water) (Hooper, 1986), kept in solution I (1 unit of glycerol and 79 unit of pure water) at 35 to 40°C for 12 hr, then solution II (5 units of glycerol and 95 units of ethanol (96%)) at 40°C for 3 hr and processed to glycerol on permanent slides, and then 10 specimens of males and females of each species were identified on the basis of their morphological characters and morphometric measurements. Morphological characters were measured under the microscope with a Leica DM 4000.

## Molecular studies

### Nematode DNA extraction

DNA extraction was performed using one to five individuals of nematode species. Nematodes were rinsed for approximately 5 min in autoclaved Milli-Q water and then transferred in a 1.5 ml micro tube with 50 µL of DNA extraction buffer (DEB) that was prepared previously and composed of 0.25 mg Proteinase K (Fisher Scientific: BP-1700-500) per 1 mL of 1 × PCR buffer (Fisher Scientific: BP6112). Nematodes were crushed using sterile micro pestle and were incubated for 2.5 hr in a water bath at 60°C, followed by 15 min incubation at 95°C to inactivate the Proteinase K. The tubes were cooled in ice for 5 min and stored at −20°C for PCR (Polymerase Chain Reaction).

### PCR and sequencing

A total of 2.0 µL DNA (from 50 µL DNA) was used as template during PCR step, together with 0.4 µL of each primer (forward and reverse), 1.25 µL dNTPs, 2.5 µL of 10 × Buffer, 2.0 µL of Titanium Taq, and 18.25 µL of H_2_O. The final volume was 25 µL. For 18S amplification, M13-18S-1-2A- (Forward primer: TGTAAAACGACGGCCAGTCGATCAGATACCGCCCTAG) and M13-18S-r2b- (Reverse primer: CAGGAAACAGCTATGACTACAAAGGGCAGGGACGTAAT) were used under initial denaturation at 94°C for 3 min, 94°C for 30 sec as denaturation, annealing at 57°C for 30 sec (40 cycles), extension at 68°C for 1 min (40 cycles), and final extension at 68°C for 3 min (40 cycles). For 28S amplification, M13 D2A-28S- Forward primer: TGTAAAACGACGGCCAGTACAAGTACCGTGAGGGAAAGT M13 D3B-28S- Reverse primer: CAGGAAACAGCTATGACTGCGAAGGAACCAGCTACTA were used following the same procedure that was applied for 28S amplification step. PCR products were purified and sequenced using Sanger sequencing method.

### Type designation and deposition

*A. stammeri* slides used for morphological studies in this manuscript are deposited at Department of Plant Protection, Cukurova University, Adana, Turkey.

## Results

### Systematics

#### Aphelenchoides stammeri

Based on morphological and molecular studies, nematodes were identified as *Aphelenchoides stammeri* (Körner, 1954) (Nematoda: Aphelenchoididae) extracted from a trap log of *Pinus nigra* taken from forest sites (328673 N and 4142474 E, ~1200 m elevation) of Sütçüler Forest Enterprise of Isparta Regional Forestry Directorate.

### Females

**Figure 1:**
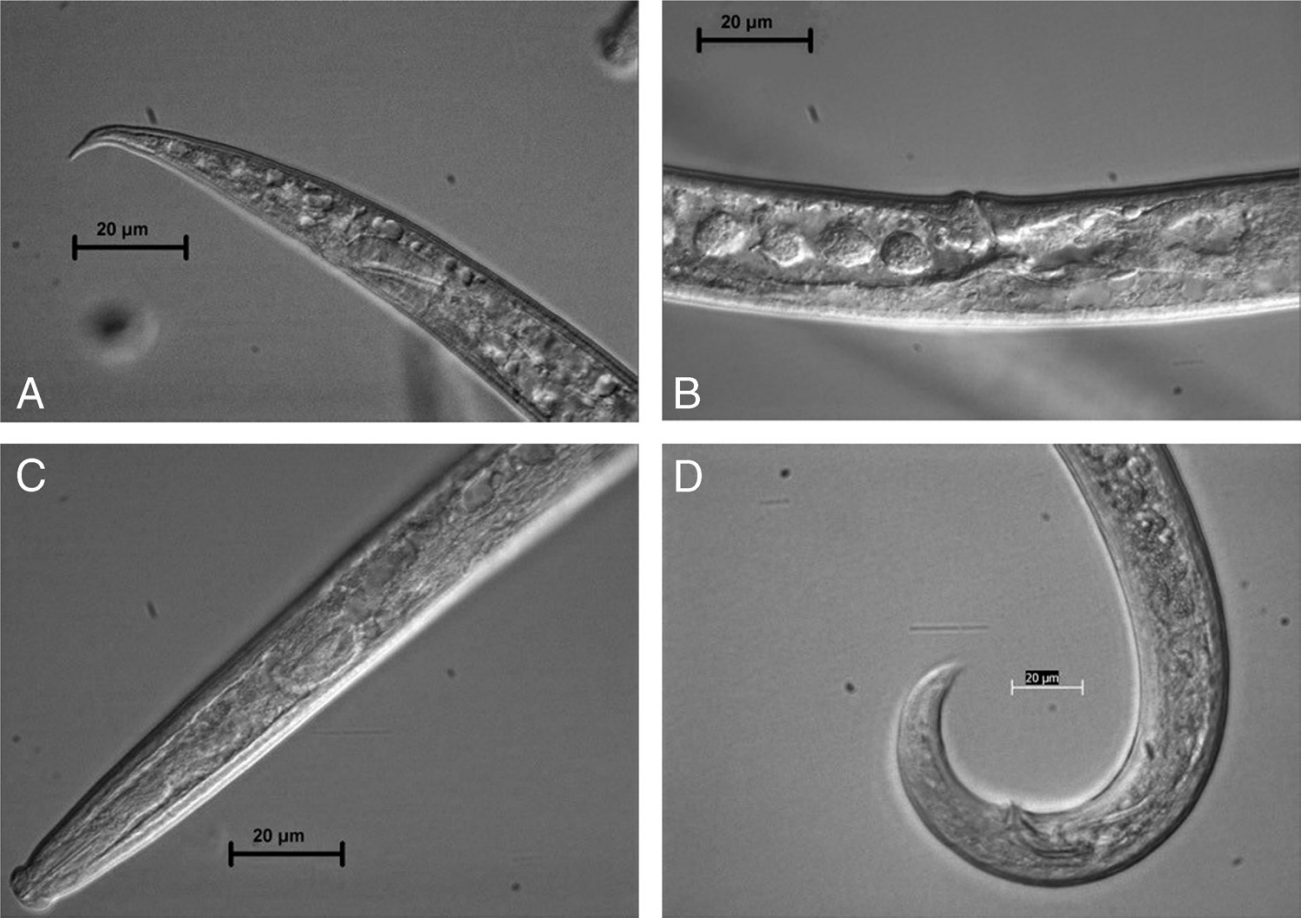
*Aphelenchoides stammeri* (A: female tail, B: female vulva, C: female anterior region and D: male tail. Scale bar = 20 µm).

Body is cylindrical, slightly ventrally arcuate when heat relaxed and fixed, 0.80 to 0.97 mm long. Cuticle with fine transverse annulations is present, and annulus is 0.9 to 1.1 µm wide (Fig. 1A-C and Table 1). Cephalic region is hemispherical in lateral view, 3.5 to 4.5 µm high and 6.5 to 7.5 µm wide, distinctly set off from body, appearing smooth and with six equal lips. Stylet is 16 to 17 µm long; conical portion is slightly longer than the cylindrical shaft, and well-developed minute stylet knobs are present. Each knob is 1.5 to 2 μm in length, with a shallow constriction in the middle, and the three knobs together are cylindrical.

**Table 1.** Measurements of *Aphelenchoides stammeri*.

Characteristics	Female (Turkish population)	Male (Turkish population)	Female (Körner, 1954; Urek et al., 2007)	Male (Körner, 1954; Urek et al., 2007)
n	10	10	6	5
L	918.7 ± 46.2 (800.0–972.8)	851.8 ± 61.8 (776.0–976.0)	777.7 ± 98.2 (673.9–905.3)	729.4 ± 53.8 (649.5–790.3)
a	31.9 ± 1.4 (30.4–33.0)	33.5 ± 5.3 (21.0–40.6)	38.3 ± 2.7 (34.9–42.3)	37.4 ± 3.5 (34.4–43.0)
b	5.7 ± 0.5 (4.8–6.7)	5.7 ± 0.4 (5.1–6.4)	Not measured	Not measured
c	14.4 ± 1.2 (13.4–16.7)	21.1 ± 1.1 (19.4–22.5)	15.7 ± 1.1 (14.5–17.8)	17.8 ± 1.4 (16.3–19.6)
c'	4.0 ± 0.3 (3.3–4.5)	2.4 ± 0.1 (2.2–2.7)	4.1 ± 0.4 (3.7–4.6)	3.2 ± 0.2 (3.1–3.5)
tail	63.8 ± 3.6 (56.0–68.8)	40.0 ± 2.0 (38.4–43.2)	49.4 ± 5.3 (44.9–59.8)	41.0 ± 2.4 (39.0–43.6)
V (%)	67.8 ± 1.6 (67.0–72.0)	–	68.1 ± 0.6 (66.9–68.5)	–
Stylet	17.1 ± 0.6 (16.0–17.6)	17.4 ± 0.4 (16.0–17.6)	15.2 ± 1.0 (14.1–16.5)	14.7 ± 1.4 (13.2–16.6)
Vulva/anus distance	230.0 ± 11.1 (214.4–240.0)	–	Not measured	Not measured
Pharynx	159.5 ± 11.7 (142.4–184)	148.8 ± 11.3 (128.0–164.8)	Not measured	Not measured
Uterus sac	146.7 ± 8.3 (131.2–158.4)	–	Not measured	Not measured
Anterior end to bulbus	80.8 ± 2.8 (76.8–84.8)	77.6 ± 2.5 (72.0–81.6)	Not measured	Not measured
Testis (%)	–	55.6 ± 6.8 (44.0–69.0)	–	Not measured
Spicule	–	20.3 ± 3.2 (12.8–24.0)	–	20.2 ± 1.4 (18.1–21.6)

Measurements in µm and in the form: mean ± SD (range).

Procorpus is cylindrical, median esophageal bulb is oval, with a distinct valvular apparatus. Spherical to rectangular median bulb is present, with crescentic valve plates, 16 to 17 µm long, 10 to 12 µm wide, located 50 to 58 µm from the anterior end. Pharyngeal glands are moderately developed and clearly visible, intestine overlaps dorsally, extending for 72 to 123 µm. Nerve ring is located 90 to 105 µm from the anterior end. Excretory pore, with 1.0 to 1.5 body widths, is posterior to nerve ring. Hemizonid is indistinct. Intestine lumen is a visible straight tube; rectum is straight. Lateral fields occupy 25 to 33% of the body diameter at mid-body region. There are four incisures in the lateral fields at mid-body. Anus has a relatively long lunate aperture.

Reproductive system is monoprodelphic, with outstretched ovary with oocytes in a single line. The length of post-uterine sac (PUS) is 4 to 5 times the anal body width. Uterus is thick walled; post-uterine sac (PUS) occupies 32 to 42% of distance from vulva to anus, and its length is approximately 2.8 to 4.2 times the corresponding body diameter. Vagina is straight, with a pronounced cuticular annulus, oval in optical section, as it joins the uterus. Vulva is a long transverse slit without vulva flap.

The shape of the tail terminus is subject to considerable variation within populations of species of the genus *Aphelenchoides*. In some species, this character may be relatively more stable and constant than others. Tail shape can be divided into four groups: (i) tail shape with no outgrowth or mucrone; (ii) tail with one or two mucrone on terminus; (iii) tail with tetramucrone on tips like spine or star shape; (iv) tail outgrowth differs than spine or star.

*A. stammeri* females have a relatively long, thin, conical and pointed tail. Conical tail is ventrally curved. The length of the tail is 3 to 4.5 times the anal body width; it is tapered gradually beyond anus.

### Males

Genital system is monorchic, testis is outstretched, with developing spermatocytes in a single line that occupies 49 to 63% of the body length. Anterior region of body and cuticle markers are similar to female (Fig. 1D, Table 1). Tail is arcuate when relaxed, not sharply curved like a hook, with a blunt terminal spine. The spicules are clumsy, strongly curved and do not have a very sharp vertex. Testis is outstretched, with spermatocytes in a row.

Rose-thorn-shaped spicule is present, with length 19 to 22 µm and a broadly rounded apex; rostrum is tiny, with triangular shape. Tail is similar in shape to that of female, but more curved.

### Differential diagnosis

This is the first report of *Aphelenchoides stammeri* from Turkey. Morphological characteristics and measurements of Turkish populations of *Aphelenchoides stammeri* are very similar to the original description of the species by Körner (1954). Confirmation of the morphological description was made by H. Braasch (pers. Comm. with H. Braasch).

### Molecular characterization

The length of sequenced 18S rRNA was 641 bp and matched with *A. stammeri* gene for 18S ribosomal RNA, partial sequence (AB368535.1) (99% identify, e-value 0.0 and 0% gaps), and the length of sequenced 28S rRNA was 618 and matched with *A. stammeri* partial 28S rRNA gene, strain Th1/1 (AM396582.1) (99% identity, e-value 0.0 and 0% gaps).

## Discussion

During the survey of potential vectors of *Bursaphelenchus* species, *A. stammeri* was found in a wood sample. In the present study with enough specimens, morphological and morphometric studies were completed for *A. stammeri*, reported for the first time from Turkey.

Genus *Aphelenchoides* Fischer is distributed widely around the world. Some of the species were listed by Hunt (1993), and he suggested that the genus needs a major revision. Between 1894 and 2017, more than 200 nominal species belonging to the genus *Aphelenchoides* have been published (Shahina, 1996; Esmaeili et al., 2017). Many are poorly described on the basis of morphological and anatomical characters alone and are difficult to identify (de Jesus et al., 2016). Difficulty in morphology-based identification of *Aphelenchoides* species due to few discriminatory taxonomic characters is conflated by poor descriptions of the numerous nominal species (Hunt, 1993; Hockland, 2001).

Most species of *Aphelenchoides* are free living and can be found in soil, decaying plant materials, galleries of wood-boring insects, etc. (Hunt, 1993). Several species have been reported as ectoparasites or endoparasites of plants. The best known species is *A. besseyi* Christie, which is the causal agent of white tip disease of rice and is a facultative ectoparasite and endoparasite on the leaves/young tissues of rice (Hunt, 1993). In the current study, we isolated *A. stammeri* from wood chips of *P. nigra* trap logs.

Plant parasitic nematodes in forest trees, including genus *Bursaphelenchus* and *Aphelenchoides*, are not well studied in Turkey. Detailed surveys and taxonomic studies should be carried out to find new species from different regions of Turkey. This study contributes to the nematode fauna of Turkey by adding one new species.
